# Coronary artery calcium score plays an important role for cardiovascular risk stratification in the statin benefit groups of asymptomatic individuals

**DOI:** 10.1186/s12944-017-0560-0

**Published:** 2017-09-12

**Authors:** Dong-Hyeon Lee, Ho-Joong Youn, Hae-Ok Jung, Kiyuk Chang, Yun-Seok Choi, Jung Im Jung

**Affiliations:** 1 Division of Cardiology, Department of Internal Medicine, Seoul St. Mary’s Hospital, The Catholic University of Korea, #505 Banpo-dong, Seocho-gu, Seoul, 137-701 South Korea; 2 Division of Cardiology, Department of Radiology, Seoul St. Mary’s Hospital, The Catholic University of Korea, #505 Banpo-dong, Seocho-gu, Seoul, 137-701 South Korea

**Keywords:** Coronary artery, Calcium, Computed tomography, Statins, Primary prevention

## Abstract

**Background:**

The purpose of this study was to describe and analyze the relationship between statin benefit groups based on statin-intensity class of drugs and coronary artery calcium score (CACS) using multidetector computed tomography (MDCT) in an asymptomatic Korean population.

**Methods:**

A total of 3914 asymptomatic individuals (mean age: 55 ± 10 years; male: female = 2649: 1265) who underwent MDCT for health examination between January 2009 and December 2012 were retrospectively enrolled. They were categorized into three groups based on statin-intensity class of drugs (high-intensity (*n* = 1284, 32.8%); moderate-intensity (*n* = 1602, 40.9%) and low-intensity (*n* = 931, 23.8%) statin therapy groups) according to the American College of Cardiology (ACC)/American heart Association (AHA) 2013 guideline and the relationship between CACS and statin benefit group was analyzed. The statin benefit group was defined as individuals who should be considered moderate- and high-intensity statin therapy.

**Results:**

Ten-year atherosclerotic cardiovascular disease (ASCVD; 12.6 ± 5.3% vs. 2.9 ± 1.9%, *p* < 0.001) and CACS (98 ± 270 vs. 3 ± 2, *p* < 0.001) were significantly higher in the high-intensity group compared to the moderate-intensity statin therapy group. In the high-intensity statin therapy group, age [odds ratio: 1.299 (1.137–1.483), *p* < 0.001], male gender [odds ratio: 44.252 (1.959–999.784), *p* = 0.001], and fasting blood glucose [odds ratio: 1.046 (1.007–1.087), *p* = 0.021] were independent risk factors associated with CACS ≥300 on multivariate logistic regression analysis.

**Conclusions:**

CACS on MDCT might be an important complementary tool for cardiovascular disease risk stratification. This study indicates that individualization of statin therapy as well as lifestyle modification will be useful in asymptomatic individuals, especially those in whom high-intensity statin therapy is required.

## Background

Although several large-scale epidemiological studies including the Framingham Heart Study have shown the importance of a balanced diet and regular exercise in maintaining good health, differences in cardiovascular risk factors between men and women and the detrimental effects of cigarette smoking are significant factors in the development of coronary heart disease (CHD), leading to angina pectoris, myocardial infarction (MI) and death [1]. However, there are some limitations in the estimation of cardiovascular morbidity and mortality risk, such as a possible overestimation in a low-risk population or underestimation in a high-risk population [2, 3].

The most recent guidelines were published in 2013 by the American College of Cardiology (ACC) and the American heart Association (AHA), and addressed the prevention of cardiovascular diseases (CVD) by better assessing cardiovascular risks and the treatment of blood cholesterol [4, 5]. Furthermore, these guidelines emphasized that more accurately identifying higher risk individuals for administration of focused statin therapy may improve the likelihood of benefit for those individuals [4, 5].

While screening for coronary artery calcium score (CACS) using multidetector computed tomography (MDCT) is not currently recommended for asymptomatic patients who are low risk (0 to 1 risk factor or a 10-year risk <10%) or high risk (CHD risk equivalent or a 10-year risk >20%) according to the Framingham criteria, it may be useful in patients with intermediate risk (more than two risk factors or a 10-year risk of 10–20%) [6, 7]. CACS has an excellent negative predictive value for excluding the presence of significant coronary artery disease [8, 9]. It also provides more important prognostic information for cardiovascular risk stratification than biomarkers like C-reactive protein [10, 11]. Therefore, CACS may play a role in patient management and the prediction of cardiovascular event incidence.

Little information is available on the relationship between CACS and the statin-intensity class of drugs in asymptomatic healthy individuals according to the 2013 ACC/AHA Guideline on the Assessment of Cardiovascular Risk and on the Treatment of Blood Cholesterol to Reduce Atherosclerotic Cardiovascular Risk in Adults [4, 5]. The purpose of this study was to describe and analyze the relationship between statin benefit groups based on statin-intensity class of drugs and CACS using MDCT in an asymptomatic Korean population according to the ACC/AHA 2013 guideline.

## Methods

### Study population and protocol

Between January 2009 and December 2012, a total of 3914 asymptomatic individuals (mean age: 55 ± 10 years; male: female = 2649: 1265) who underwent MDCT during a health check-up at the Health Promotion Center of Seoul St. Mary’s Hospital (The Catholic University, Seoul, Korea) were retrospectively enrolled.

The presence or absence of variable data (i.e., hypertension, diabetes, dyslipidemia, family history of cerebrovascular accident and smoking status) was defined as indicated above [12]. All data were obtained via questionnaire.

According to the ACC/AHA 2013 guideline, individuals who met the following criteria were included in the statin benefit groups based on statin-intensity class of drug: (i) clinical ASCVD; (ii) primary elevations in low-density lipoprotein cholesterol (LDL-C) ≥190 mg/dL; (iii) 40 to 75 years of age with diabetes with LDL-C 70–189 mg/dL; and (iv) an estimated 10-year ASCVD risk of 7.5% or higher [4, 5].

Exclusion criteria were as follows: the (i) prior coronary artery bypass graft (*n* = 4, 0.1%); (ii) prior percutaneous coronary intervention using stents (*n* = 39, 1.0%), (iii) irregular heartbeat (e.g., atrial fibrillation; *n* = 33, 0.8%); (iv) very severe obesity (body mass index ≥40 kg/m^2^; *n* = 9, 0.2%); or (v) inability to comply with breath holding instructions (*n* = 12, 0.3%). There were no patients with NYHA class II-IV ischemic systolic heart failure or with hemodialysis.

This study was approved by the Institutional Review Committee of St Mary’s Hospital, the Catholic University of Korea and was conducted in agreement with the Declaration of Helsinki (**KC17RESE0408**).

### Anthropometric parameter measurement

Each participant underwent a complete physical examination including anthropometric measurements. Height was measured to the nearest 0.1 cm with a portable stadiometer (InBody 720; Biospace Ltd., Seoul, Korea) and body weight was measured to the nearest 0.1 kg using a digital scale while patients were wearing a standardized health check-up gown. Body mass index was calculated as weight in kilograms divided by height in meters squared. Waist circumference was measured using a standardized tape by the same well-trained staff. Waist circumference was measured 1 inch above the umbilicus in a standing position. Systolic blood pressure (BP), diastolic BP and heart rate were measured using an automatic sphygmomanometer (BP203RV-II; Nippon Colin, Komaki, Japan) with subjects in a seated position after quietly resting for 10 min.

### Biochemical and hematologic parameter analysis assays

Fasting blood samples were taken the day of routine health check-up; 10 mL of blood was taken and mixed with ethylenediaminetetraacetic acid to prevent clotting. Plasma was obtained by centrifugation, frozen in liquid nitrogen and stored at −80 °C until further analysis.

The lipid profile, including total cholesterol, triglyceride, high-density lipoprotein cholesterol (HDL-C) and LDL-C levels, was measured using an enzymatic method by an automatic analyzer (7600–210; Hitachi Medical Corp., Tokyo, Japan). HbA1c was measured using a G8 HbA1c analyzer (Tosoh Corporation, Tokyo, Japan). Biochemistry including fasting blood glucose and C-reactive protein were measured using a biochemistry analyzer (7600–210; Hitachi Medical Corp., Tokyo, Japan).

### Ten-year atherosclerotic cardiovascular disease (ASCVD) risk

According to the 2013 ACC/AHA Guideline on the Assessment of Cardiovascular Risk and on the Treatment of Blood Cholesterol to Reduce Atherosclerotic Cardiovascular Risk in Adults [4, 5], 10-year ASCVD risk was defined in men and women between 40 and 79 years of age as the risk of developing a first ASCVD event (nonfatal MI or death from CHD, or fatal or nonfatal stroke) over a 10-year period in those free from ASCVD at the beginning of the period.

### Statin benefit groups based on statin-intensity class of drugs

As mentioned above, all participants (*n* = 3817: mean age: 55 ± 10 years; range 40 to 79 years) in this study were categorized into three lipid-lowering drug therapy groups based on statin-intensity class of drugs according to the 2013 ACC/AHA guideline [4, 5] used in randomized controlled trials reviewed by an expert panel: (i) high-intensity statin therapy group (*n* = 1284, 32.8%), defined as a daily dose that lowers LDL-C by at least 50% (absolute benefit of statin therapy or absolute-risk reduction for ASCVD); (ii) moderate-intensity statin therapy group (*n* = 1602, 40.9%), defined as a daily dose that lowers LDL-C by 30 to 49% (relative benefit of statin therapy or relative-risk reduction for ASCVD); and (iii) low-intensity statin therapy group (*n* = 931, 23.8%), defined as a daily dose that lowers LDL-C by less than 30% (no net benefit from statin therapy over a 10-year period). The statin benefit group was defined as individuals who should be considered moderate- and high-intensity statin therapy.

### Measurement of CACS using MDCT

We measured CACS using MDCT (SOMATOM Definition; Siemens Healthcare, Forchheim, Germany). Heart rate ranged from 43 to 75 bpm (mean, 67 bpm) during CT acquisition. Participants did not receive any additional premedication, such as β-blockers for heart rate control. Dual-source CT parameters were as follows: tube voltage = 120 kVp, gantry rotation time = 0.33 s, slice collimation = 64 × 0.6 mm, reconstruction slice width = 0.75 mm, reconstruction slice interval = 0.4 mm, kernel = B26f, field of view = 25 cm. Eighty milliliters of contrast agent was intravenously injected at 5 mL/s (Iohexol, IOBRIX INJ 300; Tae Joon Pharm. Ind. Co., Ltd., Seoul, Korea) using a dual-head power injector (CT Stellant; Medrad Inc., Indianola, PA, USA). Then, 50 mL of saline solution chaser were injected at 5 mL/s.

All post-processing examinations were performed using retrospective electrocardiogram gating. Scans were analyzed by consensus of two observers with more than 3 years of experience in MDCT imaging (YS Choi and JI Jung). CACS for vascular calcification were analyzed using syngo.CT CaScoring software (Siemens Healthcare; Forcheim, Germany).

Agatston score, standard parameter, was used as the product of the area of calcification per coronary tomographic segment and a factor rated 1 through 4 dictated by the maximal calcium x-ray density in that segment to define the quantity of coronary calcium, as described elsewhere [13]. The sum of all lesion scores for each major coronary artery was used including left main, left anterior descending artery, left circumflex artery and right coronary artery to generate the total CACS. Additionally, the volume score in cubic millimeters was used as a continuous parameter.

Of the participants with two statin benefit groups requiring more than moderate-intensity statin therapy (*n* = 2886, 73.7%), the obtained CACS values were classified into six categories: 0, normal (*n* = 1774, 45.3%); 1–10, minimal (*n* = 322, 8.2); 11–100, mild (*n* = 494, 12.6%); 101–400, moderate (*n* = 195, 5.0%); 401–1000, severe (*n* = 76, 1.9%) and >1000, very severe (*n* = 25, 0.6%) [6–8, 13].

### Statistical analysis

All data are expressed as mean ± standard deviation for continuous variables and as a frequency percentage for categorical variables. Analysis of among the three statin benefit groups based on statin-intensity class of drugs according to the ACC/AHA 2013 guideline was performed using the analysis of variance test for continuous variables and Tukey’s *b*-test as a post-hoc *t*-test for categorical data. Between the two more than moderate-intensity statin therapy benefit groups, clinical variables related to CACS using MDCT were assessed based on *Pearson’s* correlation coefficient. To identify independent factors associated with CACS ≥300, we used multiple logistic regression analysis and calculated odds ratios and 95% confidence intervals. All statistical tests were 2-tailed and *p-*values <0.05 were considered statistically significant.

## Results

### Clinical characteristics

The baseline clinical and laboratory findings among the three statin benefit groups based on statin-intensity class of drugs according to the ACC/AHA 2013 guideline are summarized in Table 1.

**Table 1:** Baseline clinical characteristics

Total = 3817	Low-intensity statin therapy (*n* = 931)	Moderate-intensity statin therapy group (*n* = 1602)	High-intensity statin therapy group (*n* = 1284)	*p-*value
Age, year	50 ± 12	53 ± 8	58 ± 8	<0.001
Gender, male, n (%)	466 (50.1)	751 (46.9)	761 (59.3)	<0.776
Hypertension, n (%)	272 (29.2)	488 (30.5)	493 (38.4)	<0.001
Diabetes, n (%)	21 (0.2)	191 (11.9)	211 (16.4)	<0.001
Dyslipidemia, n (%)	136 (14.6)	194 (12.1)	242 (18.8)	<0.001
Familial history of CVA, n (%)	121 (13.0)	304 (19.0)	500 (38.9)	0.145
Body mass index, kg/m^2^	24.1 ± 3.8	24.6 ± 3.6	25.4 ± 3.0	<0.001
Waist circumference, cm	85 ± 9	87 ± 9	90 ± 9	<0.001
Systolic BP, mm Hg	120 ± 13	122 ± 13	128 ± 14	<0.001
Diastolic BP, mm Hg	120 ± 13	72 ± 9	77 ± 10	<0.001
Heart rate, beats per minute	62 ± 8	64 ± 9	64 ± 10	0.206
Smoking status, n (%)	308 (33.1)	144 (9.0)	537 (41.8)	<0.001
Total cholesterol, mg/dL	138 ± 19	201 ± 32	209 ± 39	<0.001
Triglyceride, mg/dL	148 ± 16	106 ± 75	141 ± 86	<0.001
HDL-C, mg/dL	50 ± 14	54 ± 13	47 ± 10	<0.001
LDL-C, mg/dL	59 ± 11	122 ± 28	130 ± 35	<0.001
Fasting blood glucose, mg/dL	91 ± 14	98 ± 24	105 ± 28	<0.001
HbA1c, %	5.5 ± 0.4	5.7 ± 0.8	5.9 ± 0.9	<0.001
Fasting insulin, IU/L	8.8 ± 7.0	8.0 ± 5.2	9.1 ± 6.5	0.246
HOMA-IR,	1.9 ± 2.3	2.0 ± 1.6	2.4 ± 2.1	0.104
C reactive protein, mg/dL	0.11 ± 0.37	0.17 ± 0.56	0.18 ± 0.44	0.099
Ten-year, ASCVDS, %	2.1 ± 2.0	2.9 ± 1.9	12.6 ± 5.3	<0.001
CACS	2 ± 8	3 ± 2	98 ± 270	<0.001

Data are expressed as means ± standard deviation

*CVA* cerebrovascular accident, *BP* blood pressure, *HDL-C* high-density lipoprotein cholesterol, *LDL-C* low-density lipoprotein cholesterol, *HOMA-IR* homeostasis model assessment insulin resistance, *CAC* coronary artery calcium, *ASCVD* atherosclerotic cardiovascular disease

### Comparison of two statin benefit groups based on the statin-intensity class of drugs

**Fig. 1:**
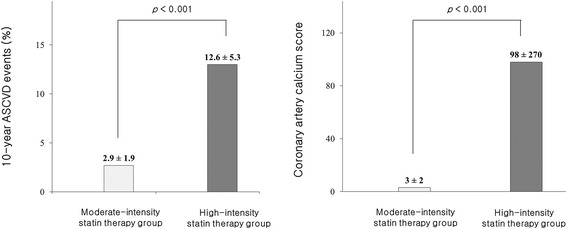
Comparison of 10-year ASCVD event and CACS between two statin benefit groups* based on statin-intensity class of drug. *Statin benefit group was defined as individuals who can be considered moderate- and high-intensity statin therapy. ASCVD: atherosclerotic cardiovascular disease; CACS: coronary artery calcium score

All laboratory findings mentioned below were significantly higher in the high-intensity statin therapy group than in the moderate-intensity statin therapy group: age (58 ± 8 years vs. 53 ± 8 years, *p* < 0.001); body mass index (25.4 ± 3.0 kg/m^2^ vs. 24.6 ± 3.6 kg/m^2^, *p* < 0.001); waist circumference (90 ± 9 cm vs. 87 ± 9 cm, *p* < 0.001); systolic BP (128 ± 14 mmHg vs. 122 ± 13 mmHg, *p* < 0.001); diastolic BP (77 ± 10 mmHg vs. 72 ± 9 mmHg, *p* < 0.001); and plasma total cholesterol (209 ± 39 mg/dL vs. 201 ± 32 mg/dL, *p* < 0.001); triglyceride (141 ± 86 mg/dL vs. 106 ± 75 mg/dL, *p* < 0.001); LDL-C (130 ± 35 mg/dL vs. 122 ± 28 mg/dL, *p* < 0.001); fasting blood glucose (105 ± 28 mg/dL vs. 98 ± 24 mg/dL, *p* < 0.001) and HbA1c (5.9 ± 0.9% vs. 5.7 ± 0.8%, *p* < 0.001); and fasting insulin (9.1 ± 6.5 IU/L vs. 8.0 ± 5.2 IU/L, *p* < 0.001) (Table 1). However, plasma HDL-C concentration (47 ± 10 mg/dL vs. 54 ± 13 mg/dL, *p* < 0.001) was significantly lower in the high-intensity statin therapy group than in the moderate-intensity statin therapy group (Table 1)**.** Ten-year ASCVD (12.6 ± 5.3% vs. 2.9 ± 1.9%, *p* < 0.001) and CACS (98 ± 270 vs. 3 ± 2, *p* < 0.001), which are important tools for risk stratification in asymptomatic individuals, were significantly higher in the high-intensity statin therapy group than in the moderate-intensity statin therapy group (Fig. 1).

### CACS in moderate- and high-intensity statin therapy groups

In the moderate-intensity statin therapy group, CACS was positively correlated with age (*r* = 0.202, *p* < 0.001) and 10-year ASCVD (*r* = 0.112, *p* < 0.001; Table 2).

**Table 2:** Correlation coefficients between CACS and clinical variables in two statin benefit groups^a^ based on statin-intensity class of drug

Total = 2886	Moderate-intensity statin therapy group (n = 1602)		High-intensity statin therapy group (n = 1284)	
	Correlation	*p-*value	Correlation	*p-*value
Age, year	0.202	<0.001	0.216	<0.001
Body mass index, kg/m^2^	0.013	0.595	0.012	0.684
Waist circumference, cm	0.026	0.299	0.041	0.151
Systolic BP, mm Hg	0.092	<0.001	0.102	<0.001
Diastolic BP, mm Hg	0.051	0.042	0.012	0.674
Heart rate, beats per minute	0.010	0.704	0.082	0.004
Total cholesterol, mg/dL	0.049	0.053	0.127	<0.001
Triglyceride, mg/dL	0.026	0.310	0.012	0.677
HDL-C, mg/dL	- 0.010	0.699	- 0.006	0.835
LDL-C, mg/dL	0.061	0.016	0.144	<0.001
Fasting blood glucose, mg/dL	0.091	<0.001	0.109	<0.001
HbA1c, %	0.097	<0.001	0.179	<0.001
Fasting insulin, IU/L	0.006	<0.001	0.012	0.709
HOMA-IR,	0.033	0.224	0.034	0.281
C reactive protein, mg/dL	0.028	0.281	0.012	0.685
Ten-year ASCVDS, %	0.112	<0.001	0.254	<0.001

^a^Statin benefit group was defined as individuals who should be considered moderate- and high-intensity statin therapy

*CAC* coronary artery calcium, *BP* blood pressure, *HDL-C* high-density lipoprotein cholesterol, *LDL-C* low-density lipoprotein cholesterol, *HOMA-IR* homeostasis model assessment insulin resistance, *ASCVD* atherosclerotic cardiovascular disease

In the high-intensity statin therapy group, CACS was positively correlated with age (*r* = 0.216, *p* < 0.001), systolic BP (*r* = 0.102, *p* < 0.001), plasma total cholesterol (*r* = 0.127, *p* < 0.001), LDL-C (*r* = 0.144, *p* < 0.001), fasting blood glucose (*r* = 0.109, *p* < 0.001), HbA1c (*r* = 0.179, *p* < 0.001), and 10-year ASCVD (*r* = 0.254, *p* < 0.001; Table 2).

### Predictability of CACS ≥300

**Fig. 2:**
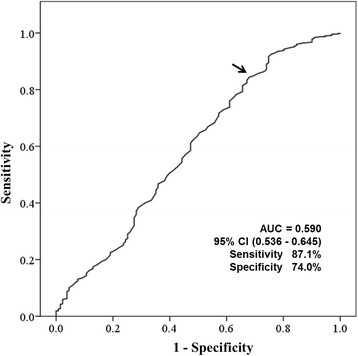
ROC curve .A 90.5 mg/dL cut-off value of LDL-C was found to have a sensitivity of 87.1% and a specificity of 74.0% for predicting the probability of CACS ≥300**.** LDL-C: low-density lipoprotein cholesterol; CACS: coronary artery calcium score

In the high-intensity statin therapy group, age [odds ratio: 1.299 (1.137–1.483), *p* < 0.001], male gender [odds ratio: 44.252 (1.959–999.784), *p* = 0.001], and fasting blood glucose [odds ratio: 1.046 (1.007–1.087), *p* = 0.021] were independent risk factors associated with CACS ≥300 on multivariate logistic regression analysis (Table 3). A 90.5 mg/dL cut-off value of LDL-C was found to have a sensitivity of 87.1% and a specificity of 74.0% for predicting the probability of CACS ≥300 (Fig. 2)**.**

**Table 3:** Multivariate logistic regression analysis for independent risk factors associated with CACS ≥300

	Moderate-intensity statin therapy group			High-intensity statin therapy group		
	Odds ratio	95% CI	*p-*value	Odds ratio	95% CI	*p-*value
Age	1.206	0.990–1.470	0.062	1.299	1.137–1.483	<0.001
Gender, male	6.726	0.149–302.635	0.326	44.252	1.959–999.784	0.017
Body mass index	0.834	0.491–1.415	0.501	1.095	0.735–1.632	0.655
Waist circumference	0.984	0.841–1.150	0.838	0.956	0.838–1.089	0.498
Systolic BP	1.015	0.901–1.144	0.801	1.066	0.999–1.138	0.053
Diastolic BP	1.027	0.884–1.194	0.723	0.988	0.889–1.097	0.816
Heart rate	0.969	0.901–1.097	0.906	0.958	0.895–1.025	0.215
Total cholesterol	1.010	0.850–1.105	0.635	1.056	0.978–1.139	0.164
Triglyceride	1.049	0.987–1.035	0.385	0.990	0.973–1.007	0.232
HDL-C	1.021	0.900–1.221	0.542	0.901	0.800–1.015	0.087
LDL-C	0.977	0.893–1.168	0.757	0.944	0.872–1.022	0.155
Fasting blood glucose	0.753	0.837–1.141	0.773	1.046	1.007–1.087	0.021
HbA1c	1.734	0.087–6.551	0.797	1.034	0.321–3.325	0.956
Fasting insulin	0.131	0.261–11.521	0.569	1.525	0.931–2.499	0.094
HOMA-IR	1.279	0.000–377.759	0.617	0.235	0.041–1.346	0.104
C reactive protein	0.879	0.809–2.024	0.292	0.249	0.008–8.009	0.432
Ten-year ASCVDS	0.948	0.386–1.999	0.758	0.967	0.831–1.127	0.669

*Statin benefit group was defined as individuals who should be considered moderate- and high-intensity statin therapy

*CAC* coronary artery calcium, *CI* confidence intervals, *BP* blood pressure, *HDL-C* high-density lipoprotein cholesterol, *LDL-C* low-density lipoprotein cholesterol, *HOMA-IR* homeostasis model assessment insulin resistance, *ASCVD* atherosclerotic cardiovascular disease

## Discussion

Recent guidelines published by the ACC/AHA in 2013 demonstrate the importance of the prevention of CVD by accurate identification of higher risk individuals for statin therapy and by prompt decision-making regarding statin-intensity class of drugs for those most likely to benefit [4, 5].

### Beneficial versus controversial effects of statin therapy

Statins are inhibitors of 3-hydroxy-3-methylglutaryl coenzyme A (HMG-CoA) reductase. The ACC/AHA 2013 guideline to reduce CVD risk, derived from systematic reviews and meta-analysis of randomized controlled trials, recommends individualization of statin therapy [4, 5] as well as lifestyle modification (i.e., adhering to a heart-healthy diet, regular exercise habits, avoidance of tobacco products, and maintenance of a healthy weight) [14]. In the present study, we found 90.5 mg/dL LDL-C to have a high sensitivity and specificity for predicting the probability of a CACS >300 on MDCT. However, the role of blood cholesterol levels in CHD and the efficacy of cholesterol-lowering statin therapy remain controversial [15, 16].

In addition, this guideline concluded that statin therapy had no benefit on CHD mortality in a high-risk primary prevention population [4, 5]. However, in recent meta-analysis of the benefits of statin, Savarese et al. showed that statin therapy reduces all-cause mortality and cardiovascular events in elderly people without established CVD [17]. Furthermore, in a meta-analysis of Cholesterol Treatment Trialists’ Collaboration, although Fulcher et al. demonstrated that statin therapy was clearly beneficial in reducing cardiovascular events [18], Abramson et al. showed no benefit in terms of mortality outcomes [19].

### Diabetes is associated with statin therapy in asymptomatic individuals

Diabetes, as a CVD “risk equivalent” and considered more aggressive lipid-lowering therapy, is associated with a marked increase in the risk of cardiovascular events, leading to MI, stroke and CHD-related death [12].

Nevertheless, in a multicenter, observational study, Dormuth et al. suggested that higher potency statin treatment is associated with increased risk of new-onset diabetes compared to lower potency statin treatment [20]. Although meta-analyses of clinical trial data revealed that increased risk of new-onset diabetes associated with statin therapy and statin use prior to diagnosis of new-onset diabetes do not increase the prevalence of microvascular disease, Betteridge et al. emphasized that statin-treated patients at high risk of developing diabetes should be monitored regularly for changes in blood glucose and HbA1c levels [21].

The present study demonstrated that diabetes is an independent risk factor associated with CACS ≥300 on MDCT and plays an important role in coronary artery calcification in asymptomatic middle-aged individuals, especially in those requiring high-intensity statin therapy. Further investigation is needed in larger populations via multicenter trials to predict major adverse cardiovascular events and establish the role of diabetes as a cardiovascular disease “risk equivalent” in asymptomatic individuals.

### Limitations

Our study has several limitations. In particular, the following elements were omitted: (i) cost-benefit evaluation of statin therapy; (ii) additional consideration of other factors such as apolipoprotein B, glomerular filtration rate, microalbuminuria, ankle-brachial index and carotid intima-media thickness; (iii) subgroup analysis such as racial, regional and gender differences; and (iv) analysis of secondary prevention in patients with established ASCVD.

## Conclusion

In summary, CACS using MDCT might be an important complementary tool for CVD risk stratification. This study indicates that individualization of statin therapy as well as lifestyle modification will be useful in asymptomatic individuals, especially those in whom high-intensity statin therapy is required. In addition, further research on clinical outcomes is needed.

## Abbreviations

ACC: American College of Cardiology
AHA: American heart Association
ASCVD: Atherosclerotic cardiovascular disease
BMI: Body mass index
BP: Blood pressure
CACS: Coronary artery calcium score
CHD: Coronary heart disease
CI: Confidence intervals
CVA: Cerebrovascular accident
CVD: Cardiovascular disease
HDL-C: High-density lipoprotein cholesterol
HOMA-IR: Homeostasis model assessment insulin resistance
LDL-C: Low-density lipoprotein cholesterol
MDCT: Multidetector computed tomography
MI: Myocardial infarction
